# Aviation carbon transfer and compensation of international routes in Africa from 2019 to 2021

**DOI:** 10.1038/s41597-023-02219-7

**Published:** 2023-05-19

**Authors:** Qiang Cui, Bin Chen

**Affiliations:** School of Economics and Management, Southeast University, Nanjing, 211189 China

**Keywords:** Environmental economics, Governance, Governance

## Abstract

As an underdeveloped region, the aviation industry in Africa is developing rapidly, and its carbon emissions play an essential role in achieving carbon neutrality in the aviation industry in underdeveloped areas. However, the problem of carbon transfer caused by passenger flow on international routes has not been addressed, especially in Africa. This paper first calculates the CO_2_ emissions of African international routes from 2019 to 2021 based on the Modified Fuel Percentage Method (MFPM) and the ICAO standard methods. Then, we measure African routes’ carbon transfer and carbon compensation. The most carbon transfer routes between African countries and from countries outside Africa to African countries are from Ethiopia to Kenya and from Honduras to Ghana. Relatively poor countries bear a significant amount of carbon transfer.

## Background & Summary

Conducting in-depth research on African emissions data is vital to the international civil aviation industry. First, in 2021, the average per capita GDP of 54 African countries was 5,792.73 Geary-Khamis dollars, 27.86% of the world average^1^. As an underdeveloped region, Africa’s airlines use many old aircraft, resulting in a low utilization rate of jet fuel and ineffective emissions^2,3^. Secondly, the African aviation industry has developed rapidly in recent years, occupying a significant global position. The African aviation industry’s average air passenger volume growth rate during 2010–2019 (before COVID-19) was 8.45%1. Finally, the average proportion of service trade in the GDP of African countries in 2010–2019 has always been more significant than 15%^1^. The aviation industry is an essential part of service trade, so the aviation industry is one of the pillar industries for African economic development. Therefore, studying the issue of aviation emissions is crucial for African economic development.

There is currently significant research interest in reducing aviation’s climate impact^4,5^. Therefore, it is of practical significance to study aviation carbon transfer and carbon compensation. First, the existing aviation emission accounting is mainly based on routes or airports, with no specific carbon emission responsibility. Therefore, the study of carbon transfer is conducive to clarifying the responsibility of airlines and countries for carbon emission reduction and promoting the reduction of aviation carbon emissions; In addition, many countries are passively receiving aviation carbon emissions, and the development of the aviation industry is crucial to their economic development. Therefore, if countries receiving carbon emissions can get some carbon compensation, it is conducive to promoting the development of their aviation industry, thus promoting economic growth.

Climate change is one of the most concerning environmental problems in the world today, and the greenhouse gas dominated by CO_2_ is the leading cause of global warming^6,7^. The study found that aviation emissions accounted for 20% of the worldwide carbon footprint of tourism^8^. Therefore, measuring aviation carbon emissions provides empirical data for research on aviation carbon emissions, which is the basis for research on aviation carbon emissions. There are three commonly used aircraft emission calculation methods. The first method is EEA/IPCC approach^9,10^ (including Tier-1, Tier-2, and Tier-3), which calculates aircraft carbon emissions based on fuel consumption during the CCD (Climbing/Cruising/Descending) and LTO (Landing and Take-off) stages. This method considers the emission characteristics of aircraft from the fuel perspective, ignoring the differences between engine types^11^. The second method is the UK Department for Environment, Food and Rural Affairs (DEFRA) method^12^. DEFRA method takes the product of distance and emission coefficient as the carbon dioxide emission equivalent, ignoring the differences between different aircraft types. The third method is International Civil Aviation Organization (ICAO)approach^13–15^, which is more suitable for calculating aircraft emissions during the LTO (Landing and Take-off) phase^16–19^. It does not consider the differences between different types of aircraft. Therefore, the carbon emission in this paper is divided into the CCD and LTO stages. The carbon emission in the LTO stage adopts ICAO standard method, and the carbon emission in the CCD stage adopts the MFPM method^20–22^. The technique adopted in this paper considers more detailed models and carbon emission intensity at different distances, which improves the calculation accuracy.

Scholars have done little research on carbon emissions transfer and carbon compensation in the aviation industry. The existing research about carbon emissions transfer is mainly based on international trade processes and resource flows^23–26^. Early research on carbon transfer focused on carbon transfer between countries based on trade^27^. With the gradual deepening of research, the research on carbon transfer in various regions within a country was gradually increasing^28–32^. As an essential way to promote the coordinated and sustainable development of the industry, the carbon compensation mechanism is a new field of ecological compensation developed in the context of global climate change and low-carbon development^33,34^. Scholars have studied carbon compensation from many aspects. Xia and Yang looked at the temporal and spatial changes of the carbon compensation zones of 157 county-level units in the Beijing Tianjin Hebei urban agglomeration from the perspective of major functional areas^35^. Wang *et al*. also studied the temporal and spatial differences in carbon emissions and inter-regional carbon compensation in major functional areas of Guangdong Province, China^36^. Scholars have studied carbon compensation based on land use change^37,38^. The scholars pointed out that the carbon compensation mechanism is conducive to stimulating low-carbon agricultural production behaviour with positive externalities and reducing agricultural carbon emissions^39^.

To sum up, few scholars have studied aviation carbon emissions in Africa. Africa’s aviation industry should be paid more attention to as an underdeveloped region. Whether countries undertaking more carbon transfer should be compensated accordingly is critical for underdeveloped African countries. Furthermore, the current research on carbon emission transfer is mainly based on the international trade process and resource flow, lacking research on the carbon transfer of the aviation industry. Existing research on carbon compensation is mostly the overall carbon compensation between countries or regions, and the research on carbon compensation in the aviation industry is insufficient. This paper attempts to study carbon transfer and compensation based on the data of African international routes, which renews carbon emission transfer and carbon compensation research fields and provides some suggestions for promoting the emission reduction of aviation carbon in Africa.

## Methods

### Collect detailed flight information

Before the carbon emissions are calculated, we need to collect detailed flight information, which contains two parts: the weekly route information and the weekly flight information. For the weekly routes, we need to log on to the website of Variflight^40^ (https://map.variflight.com/), click “query”, and enter “departure country” and “arrival country”. Then we can get the route information between the two countries and query all African countries to get all the international routes in Africa (mainly the origin and destination airport). After getting the route information, we open the app of Variflight with the mobile phone and record all the flight information (including frequency, aircraft type, aircraft seat layout, flight time, flight distance, transit information, etc.). Then we collect the data for all the routes. This part is the most time-consuming.

**Fig. 1:**
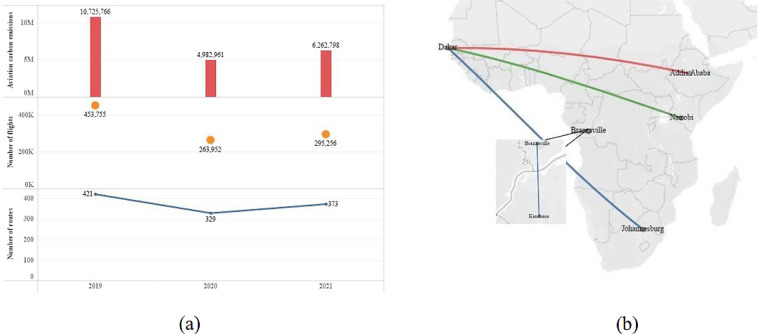
Statistical characteristics of the air routes. (**a**)Aviation carbon emissions, number of flights, and number of routes. (**b**) The farthest and nearest route during 2019–2021. (The blue, red and green lines represent 2019, 2020 and 2021, respectively).

This paper collects the route information of countries in the African region, and the specific information is shown in Fig. 1. As shown in Fig. 1a, the African routes have been affected by COVID-19 from 2019 to 2021. The number of routes, flights, and carbon emissions has declined due to COVID-19, while the number of airlines has increased gradually in the past three years, from 2019 to 2021. The number of routes dropped from 421 in 2019 to 329 in 2020 and recovered to 373 in 2021, but there was still a significant gap from 2019. The number of flights reduced from 453,755 in 2019 to 263,952 in 2020 and then increased to 295,256 in 2021. As shown in Fig. 1b, The shortest routes from 2019 to 2021 were 26 km from Brazzaville to Kinshasa. The farthest routes significantly differ in 2019, 2020, and 2021. In 2019, the longest route was from Johannesburg to Dakar, with a length of 6,685 km. In 2020, the longest route was 6,095 km, from Addis Ababa to Bryce Diane. In 2021, the longest route was from Nairobi to Dakar, 6,377 km.

### Calculate the emissions of the LTO stage (ICAO standard method)

First, we must log on to the ICAO Aircraft Engine Emissions Databank (https://www.easa.europa.eu/domains/environment/icao-aircraft-engine-emissions-databank) and download the latest emission databank (including the carbon emission data of all types of engines in an LTO cycle). Second, we build an engine database for each aircraft we collect from Variflight (including engine type and the number of engines). Finally, we can get the LTO emissions by multiplying by the flight frequency and accumulating.

### Calculate the emissions of the CCD stage

This part contains two steps: Calculate the emission intensity of the CCD stage and calculate the emissions in the CCD stage. For the first one, we need to divide the distance segments and then calculate the *ratio*_*cr*_ for each aircraft in different distance segments. Then we get the CO_2_ emission intensity of each aircraft in each distance segment. The emission intensity of each aircraft type is multiplied by the flight distance, flight frequency, and other data to obtain the emission at the CCD stage.

The CCD emissions *E*(*Q*) can be calculated by

1 $$\begin{array}{ccl}{E}_{j}(Q) & = & {I}_{j}\times F(Q)={I}_{j}\times {M}_{fuel}\times weight(Q)={I}_{j}\times (1-{M}_{ff})\times weight(Q)\\  & = & {I}_{j}\times \left(1-{\prod }_{i=1}^{n}\frac{{W}_{i}}{{W}_{i-1}}\right)\times weight(Q)={I}_{j}\times \left[1-{e}^{-\frac{dis\times rati{o}_{cr}}{10\times v}}\right]\times weight(Q)\\  & = & {I}_{j}\times \left[1-{e}^{-\frac{dis\times rati{o}_{cr}}{10\times v}}\right]\times (aircraftbareweight+100\times (load\;factor\times number\;of\;seats)+50\times seat)\end{array}$$

*I*_*j*_ is the emission coefficient of pollution j of aviation kerosene^41^. *weight*(*Q*) is the total weight of the aircraft. *M*_*fuel*_ is the fuel coefficient, ${M}_{ff}={\prod }_{i=1}^{n}\frac{{W}_{i}}{{W}_{i-1}}$ is a fuel weight proportionality coefficient, which is usually calculated by Fuel Percentage Method (FPM)^20–22^. The total sections of a whole flight contain seven task sections: Engine Starting, Taxiing, Taking Off, Climbing, Cruising, Descending and Landing. *W*_*i*_/*W*_*i-*1_ as the fuel weight proportionality coefficient of task section *i* (*i*=1, 2, …, 7). *number of seats* is certified seat number, *seat* is the actual passenger number.

As we only consider the CCD section in this study, so we define the *W*_*i*_/*W*_*i-*1_ of other sections is 1. The *W*_*i*_/*W*_*i-*1_ of Climbing and Descending are 0.980 and 0.990. The equation of the CCD section to calculate *W*_*i*_/*W*_*i-*1_ is ${W}_{i}/{W}_{i-1}={e}^{-\frac{dis\times {c}_{cr}}{10\times v\times L{D}_{cr}}}$. *dis* is the cruising distance, *v* is the cruising speed, *c*_*cr*_ is the fuel consumption ratio when the aircraft is cruising, *LD*_*cr*_ is the lift-drag ratio when the aircraft is cruising. The value of *c*_*cr*_ and *LD*_*cr*_ has direct relationships with the aircraft type. We define $rati{o}_{cr}=\frac{{c}_{cr}}{LD{c}_{cr}}$, and then for the cruising task section, the *W*_*i*_/*W*_*i-*1_ is ${W}_{i}/{W}_{i-1}={e}^{-\frac{dis\times rati{o}_{cr}}{10\times v}}$.

The actual flying time of each flight is applied to check the results of *ratio*_*cr*_, and get the emission intensity.

For CO_2_, the emission coefficient is fixed, which is *I*_*CO*2_=3.157 *kg*/*kg*.

### Calculate the total emissions

The LTO and CCD emissions are added to obtain the total emission. Affected by COVID-19, the carbon emissions dropped significantly, from 10.73 million tons in 2019^42^ to 4.98 million tons in 2020^43^, then recovered to 6.26 million tons in 2021^44^.

### Calculate the carbon transfer

**Fig. 2:**
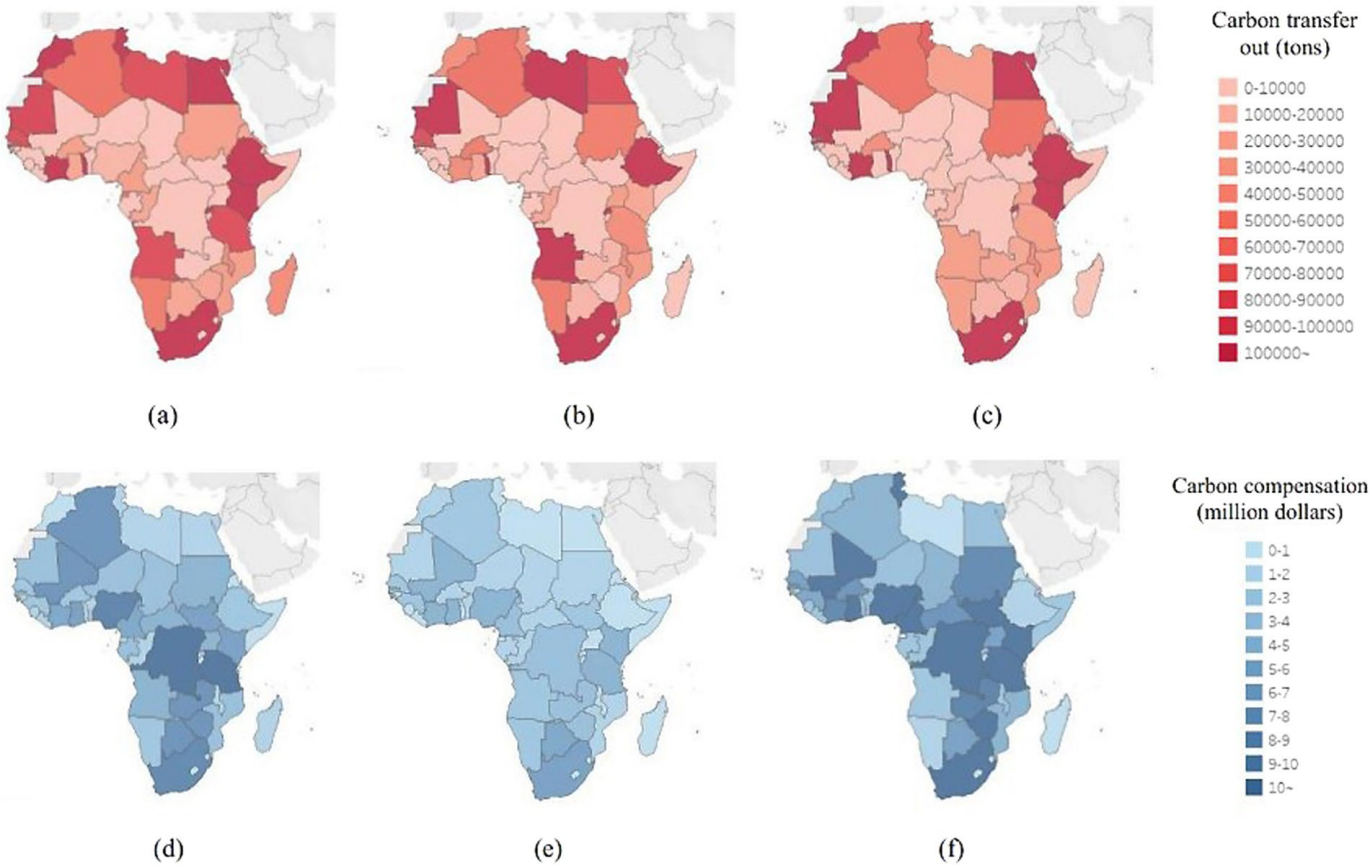
Carbon emissions transfer out and carbon compensation in African countries during 2019–2021. (**a**–**c**) show the carbon emissions transfer out in African countries from 2019 to 2021. (**d**–**f**) reveal the carbon compensation in African countries from 2019 to 2021.

**Fig. 3:**
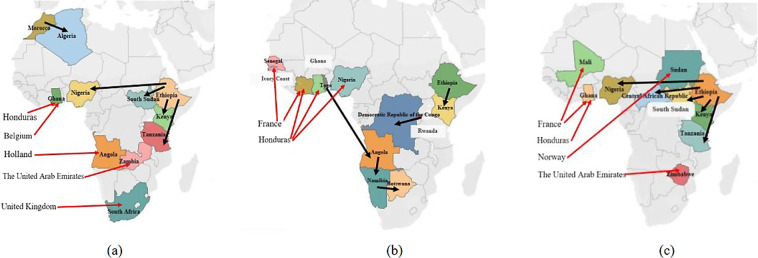
Major carbon emissions transfer paths from 2019 to 2021. (**a**–**c**) show the five largest intra African and extra African transfer paths of carbon emissions in 2019, 2020 and 2021. The black line represents the intra African transfer path, and the red line represents the extra African transfer path.

Existing studies have adequately applied the multiregional input-output (MRIO) model to calculate the international and domestic carbon emissions transfer from both the producers’ and consumers’ perspectives^45–48^. However, few scholars have studied aviation carbon transfer, so we cannot draw more inspiration from it. Our method of calculating carbon transfer is based on a combination of specific flight information and geographical distance. We divide the route according to the distance to get the length of the route in different countries and then split the emissions in the CCD stage according to the distance segment. At the same time, the emissions in the LTO stage are divided equally from the origin and destination airports. If there is a transit, the transit airport shall bear the emissions of one LTO, and the origin and destination airports shall share the emissions of another LTO equally. The distance segment is divided by the ranging function of Baidu Maps (the route has a 3-D distance). Aviation emissions are mainly completed by airlines, and airlines have corresponding nationalities, so carbon transfer between countries can be determined accordingly. For example, Qatar Airways is owned by Qatar, and its emissions in various African countries are considered Qatar’s emissions in these countries. Using the above method, we calculate the carbon transfer of the African route from 2019 to 2021^49–51^. Figure 2a–c show the carbon transfer of African countries caused by African international routes during 2019–2021. Figure 3 shows African international routes’ primary carbon emissions transfer paths from 2019 to 2021.

### Calculate the overall carbon compensation

**Fig. 4:**
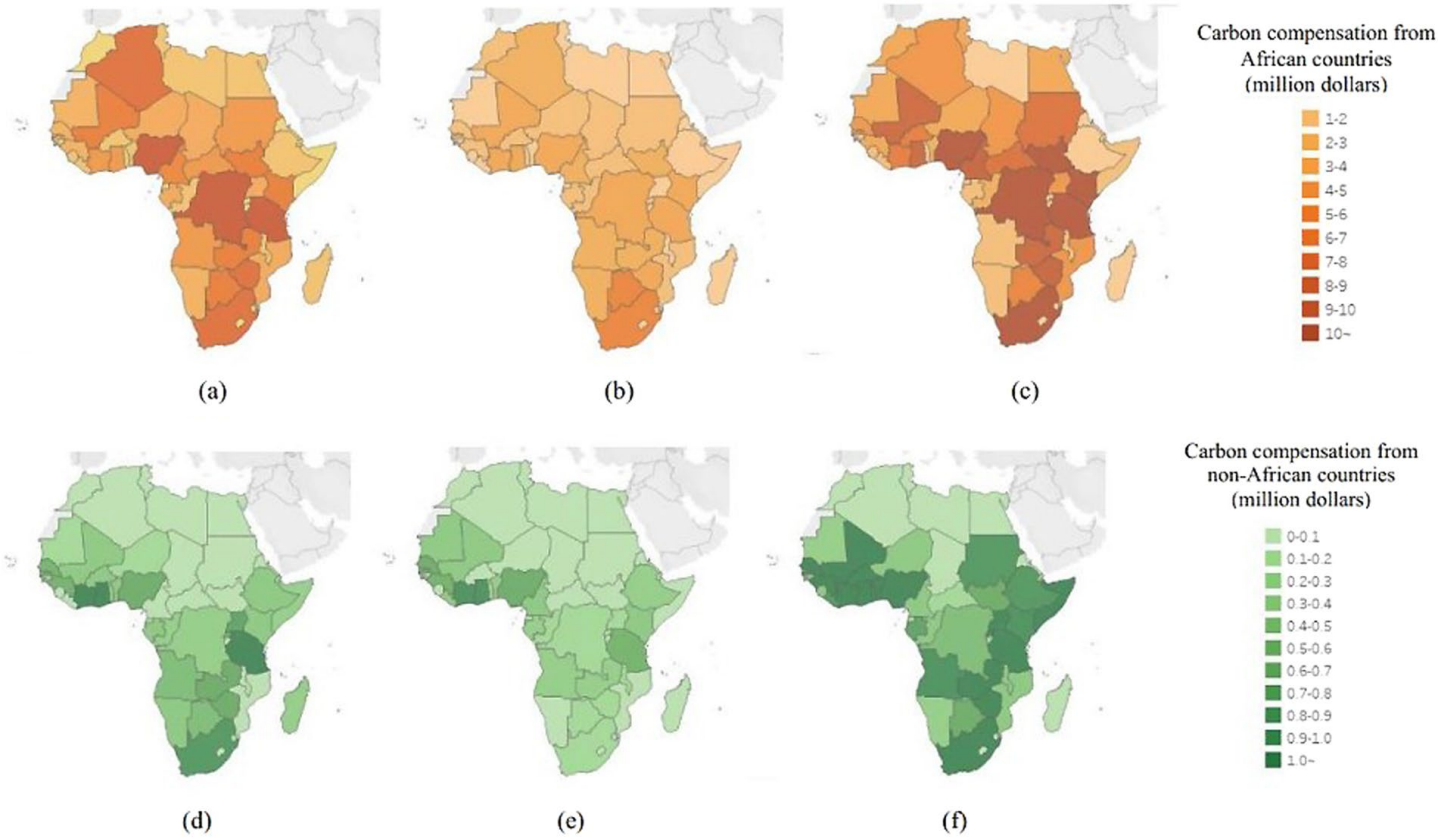
Carbon compensation from African countries and non-African countries during 2019–2021. (**a**–**c**) show the carbon compensation (from African countries) of African countries in 2019, 2020, and 2021. (**d**–**f**) show carbon compensation (from non-African countries) of African countries in 2019, 2020, and 2021.

Carbon prices for 2019, 2020, and 2021 were 23.78 dollars/ton, 27.84 dollars/ton, and 56.80 dollars/ton1. Therefore, we can calculate the country’s annual carbon compensation amount according to the formula that the carbon price multiplied by the carbon emissions transfer amount equals the carbon compensation amount. Figure 2d–f reveal the carbon compensation in African countries from 2019 to 2021. Figure 4a–c show the carbon compensation (from African countries) of African countries in 2019, 2020, and 2021. Figure 4d–f) show carbon compensation (from non-African countries) of African countries in 2019, 2020, and 2021.

## Data Records

Our calculation results are recorded in six files^42–44,49–51^. “The CCD and LTO emissions of each route and airline from the international routes in Africa in 2019. xlsx” records aircraft emissions from African international routes in 2019. The first column of this file is the serial number. The second, third, fourth, and fifth columns indicate the country and city where a route started and arrived, and the sixth and all subsequent columns indicate the emissions of each airline (including CCD stage emissions and LTO stage emissions). “The CCD and LTO emissions of each route and airline from the international routes in Africa in 2020. xlsx” and “The CCD and LTO emissions of each route and airline from the international routes in Africa in 2021. xlsx” are similar to “The CCD and LTO emissions of each route and airline from the international routes in Africa in 2019. xlsx”. “The overall carbon transfer of African air routes in 2019. xlsx” shows the carbon transfer of African international routes in 2019, the first column of this file is the countries with carbon transfer out, the second column is the category of carbon transfer out countries, the third column is the countries receiving the carbon transfer and the fourth column is the amount of carbon emissions transfer. “The overall carbon transfer of African air routes in 2020. xlsx” and “The overall carbon transfer of African air routes in 2021. xlsx” are similar to “The overall carbon transfer of African air routes in 2019. xlsx”.

## Technical Validation

### Accuracy analysis

In this section, we discuss the accuracy of the results. We have not found direct data on international routes in Africa, and we can only deduce it through indirect data. According to flight frequency, aircraft type, flight distance, aircraft weight, and other data, we calculated that the total turnover of international routes in Africa in 2019 was 12.13 billion ton-km. According to the Civil Aviation Administration of China, the fuel consumption per ton-km is about 0.29–0.32 kg/ton-km^52^. Multiplied by the emission coefficient of 3.157, the emission of African international routes in 2019 was about 11.11 million tons-12.26 million tons. Our calculation result is 10.73 million tons, the error rate is 3.57%–14.3%, and the average error is 8.93%. Considering the errors in the statistical process, the accuracy of the calculation method in this paper is relatively high. Furthermore, since our data is accurate for each airline and route, using our method to calculate carbon emissions is meaningful.

### Comparisons with existing emission databases

Since there are few databases on carbon emissions from the African aviation industry, the data in this article supplement existing data. Furthermore, the data in this article are accurate for the carbon emissions of each airline on each route, and the data scale is more accurate.

## Supplementary information

The CCD and LTO emissions of each route and airline from the international routes in Africa in 2019

The CCD and LTO emissions of each route and airline from the international routes in Africa in 2020

The CCD and LTO emissions of each route and airline from the international routes in Africa in 2021

The overall carbon transfer of African air routes in 2019

The overall carbon transfer of African air routes in 2020

The overall carbon transfer of African air routes in 2021

## Data Availability

The calculation of data is mainly done by Excel 2017, and there is no special code.
